# Southern Medical Reports

**Published:** 1851-01

**Authors:** 


### ARTICLE III.

*Southern Medical Reports*, Edited by E. D. Fenner; Volume 1st, 1849—New Orleans: B. M. Norman, 16 Camp St.

Although this volume has been already briefly noticed in this Journal, yet its merits require a more extended examination. Two leading objects seem to have induced the editor to engage in the enterprise, of which this volume is the first result. The first object was to arouse his southern brethern to greater activity and zeal in the cultivation of Medical Literature and Science; the second, to attract more attention to the very important subject of medical topography as connected with the character and prevalence of particular diseases; and judging from the contents of the volume before us, we think both are in a fair way of accomplishment. In relation to the topography and diseases of particular localities we find six articles, viz; one concerning New Orleans by the Editor; the Parish of St. Mary, La., by James B. Dungan, M. D.; Madison County, Alabama, by J. Y. Bassett, M. D.; Middle Georgia, by E. M. Pendleton, M. D.; Jackson, Miss., by S. C. Farrar, M. D.; and concerning San Antonio, Texas, by J. J. B. Wright, M. D., Surgeon U. S. A. The first of these occupies near forty pages, and in addition to a brief account of the topography and climate of New Orleans, it contains a pretty full record of the meteorology, diseases, and mortality for the year 1849; and though an interesting and instructive paper, it contains little that can be eithe[illegible] cted or condensed for the pages of this Journal.

The following conclusions in regard to the interesting subject of acclimation, may correct some very wrong impressions and we therefore quote them entire, viz;

"1st. That persons coming from more northern latitudes to this, have to undergo an *acclimation* or *seasoning*, before they become secure in the enjoyment of health.

"2d. That this acclimation may be attained without sickness; but that, most generally, it requires the endurance of one or more spells of the customary endemic fevers.

"3d. That an attack of the endemic Yellow Fever effects greater security against subsequent attacks, than any form of fever seen in the country, but that the remark is applicable, in some degree, to all of them, excepting the ordinary mild intermittents.

"4th. That persons may have the yellow fever *more* than *once*, though it is evident that those who have had one plain attack, usually have little or nothing to dread from subsequent attacks.

"5th. That *Creoles*, or natives of New Orleans, may have yellow fever, though generally, they have it in a very mild form."

Dr. Fenner tells us what is but too true of almost all the cities in the south and west, viz: that "little or nothing has ever yet been done *directly* or *expressly* with the the view to improving the sanitary condition of New Orleans." And again he says, "it will be seen by these reports, that the sanitary condition of the city is still very bad, and the mortality very great." A statement fully borne out by the tables of mortality which foot up no less than 8,809 deaths during the year, or mare than one out of every twenty of the entire population.

The article of Dr. Dungan concerning the topography and diseases of the Parish of St. Mary, La., is very brief, imperfect, and contains nothing to elicit comment.

Dr. Bassett's report on the topography, climate and diseases of Madison County, Alabama, occupies twenty-five pages; and though written in a style somewhat open to critisism contains many interesting and very valuable facts. We have space, however, to allude to those only of most direct practical application. Dr. Bassett imforms us that the endemics of Madison Co., consist chiefly, "of billious, intermitting and remitting fevers, during the summer and fall; continued and typhoid cases of the same, together with inflamation of the thoracic viscera in the winter and spring." Concerning the use of very large doses of Quinine in these diseases, the writer says: "Our physicians do not now use as large doses of Quinine as formerly; these enormous doses, 100 grs., during the apyrexia, were introduced by Dr. Fearn, in a few specific cases, after anxious consideration, and were continued in the most impirical and thoughtless manner, by physicians and families, and of course without a corresponding result." Dr. B. relates the case of a girl who took no less than 140 grs. of Quinine during one intermission. The effects are detailed in his own peculiar style as follows, viz: "I saw her at midday, she had no chill; her surface was clammy and damp, like the back of a frog; she was deprived first of hearing, then of vision, then of feeling, and lastly of speech; candles and pistols were flashed and shot about her head, aud pins stuck in her. I dashed two or three buckets of cold water on her suddenly, ordered warm stimulating enemata, and wrapped in warm blankets. In the course of the night she felt a pin scratch, at length her eyes followed a candle and she spoke; in a few days her hearing was restored. I have not had occasion to give such large doses of Quinine; 15 to 25 grs. I find sufficient."

We learn from this report, that Madison County contains about 30,000 inhabitants, who consume about 50 lbs. of Calomel, and 1000 ozs. of Quinine, annually. There are also 30 physicians in the county, whose aggregate annual income amounts to about $30,000, or one dollar per head for the entire population. Speaking of the unusual frequency of adynamic cases of disease during the past year, Dr. Bassett intimates that the extensive and prodigal use of Quinine may have contributed to this tendency. He says: "The enormous amount of Quinine that has for the past fifteen or twenty years been consumed in this neighborhood, must have wrought an impression on the constitutions of those most subject to its influences. The following paragraph may also afford a useful hint to practical readers. "Numbers of cases have lately presented themselves of habitual chills, recurring every second, third or fourth day, which have required very large doses of Quinine to check, and which returns almost every eighth or tenth day. Since August, I have treated eight successive cases of this description, (in every one of which Quinine had failed,) with opium and plasters made of mntton suet and turpentine, or cloths dipped in warm turpentine, and applied to the pit of the stomach a half hour before the expected chill. In all these cases but one, the first chill was checked, and there was no return. Although opium seems to have an excellent effect in those cases in which Quinine had failed, I have sometimes succeeded with the hot turpentine cloths alone."

The fourth article, to which we have alluded, written by Dr. E. M. Pendleton, on the topography, climate, and diseases of Middle Georgia, occupies a little more than twenty pages; and is truly a valuable paper. Perspicious, easy, and correct in its style; happy in its arrangement, and rich in its facts, it may well be characterized as a model paper of its class. Its intrinsic merits, indeed, renders it difficult either to condense its matter or extract detached paragraphs from its pages. By a happy arrangement of tabular statistics in regard to the thermometic, barometic, hygrometic facts, with coincident prevalence of diseases, and a lucid description of the physical geography of the subject under consideration, the reader is presented at once, with a highly satisfactory view of the subject. Dr. Pendleton's remarks in regard to the Etiology of Remitting and Intermitting Fevers are interesting, and strongly corroberative of the old idea that the combined influence of heat, moisture, and vegetable matter, generates a specific cause, productive of these diseases.

In regard to the comparative prevalence of disease among the two races, Dr. Pendleton's tables and observations show, that the whites are far more susceptible to diseases of the digestive organs, while pulmonary affections are greatly more prevalent among the blacks. The whites also suffer a little more frequently from indiopathic fevers, especially the male sex. These facts are doubtless easily explained by reference to the difference in the habits and natural temperament of the two races. N. S. D.

*(To be Continued.)*

